# Adaptive pathways for multimodal community-based detection of cognitive impairment: the CogScreen I study

**DOI:** 10.1038/s41598-026-61868-x

**Published:** 2026-07-23

**Authors:** Carolin Kurz, Nikola Clara-Sophie Wüsten, Paulina Tegethoff, Marleen Taute, Manolo Kehrls, Anna Hufnagel, Sylvia de Jonge, Amy Deckert, Monica Zigman Suchsland, Alexandra König, Elisabeth Feustel, Pia Heindl, Soeren Mattke, Robert Perneczky

**Affiliations:** 1 Technical University of Munich, TUM School of Medicine and Health, Department of Psychiatry and Psychotherapy, TUM University Hospital, Munich, Ismaninger Str. 22, 81675 Munich, Germany; 2 Department of Psychiatry and Psychotherapy, LMU Hospital, LMU Munich, Munich, Germany; 3 Davos Alzheimer’s Cooperative, Wayne, PA USA; 4 Ki:Elements GmbH, Saarbrücken, Germany; 5 Johanniter-Unfall-Hilfe E.V., Munich, Germany; 6 Department of Public Health (Gesundheitsreferat), City of Munich, Munich, Germany; 7 The USC Brain Health Observatory, USC Dornsife, Los Angeles, CA USA; 8 German Center for Neurodegenerative Diseases (DZNE), Munich, Germany; 9 Munich Cluster for Systems Neurology (SyNergy), Munich, Germany; 10 Ageing Epidemiology (AGE) Research Unit, School of Public Health, Imperial College, London, UK; 11 International Registry for Alzheimer´S Disease and Other Dementias (InRAD) Foundation, Deventer, The Netherlands

**Keywords:** Dementia, Alzheimer’s disease, Public health, Holistic health, Alzheimer disease / diagnosis, Dementia / diagnosis, Cognitive dysfunction / diagnosis, Early diagnosis / methods, Biological markers / blood, Speech analysis, Community health services, Primary health care, Feasibility studies, Biomarkers, Health care, Medical research, Neurology, Neuroscience

## Abstract

Early detection of cognitive impairment is essential for dementia prevention and timely care. However, implementation in primary care and community settings remains limited. Building dementia-prepared health systems requires scalable and adaptive pathways that integrate subjective, digital and biological indicators, while accounting for heterogeneity in risk, education and age. CogScreen I was a cluster-randomized trial conducted in Munich senior centers from March 2023 to March 2024. Recruitment focused on community-dwelling adults aged ≥ 60 years reporting subjective cognitive concerns. Centers were randomized to: (A) the Subjective Cognitive Decline Questionnaire (SCD-Q) only, (B) SCD-Q plus digital cognitive testing, or (C) SCD-Q plus digital testing plus blood biomarkers (Aβ1-42/1–40, pTau181, GFAP, NfL). The primary endpoints were feasibility and acceptability, assessed through structured questionnaires and follow-up interviews with participants and general practitioners. Secondary endpoints examined latent cognitive structures and biomarker associations. Exploratory factor and clustering analyses revealed multimodal subgroups across subjective, digital, and biological measures, which informed a hypothesis-generating three-tier adaptive detection framework (low, medium, high intensity). Among the 473 participants (mean age 74.1 ± 7.6 years; 66% female; 63% with tertiary education), both feasibility and acceptability were high: Participants described the assessments as personally relevant, clearly communicated, and medically meaningful, and appreciated the added value of digital testing and biomarkers. Digital cognitive testing and biomarkers captured distinct latent dimensions of learning/working memory, psychomotor attention, and glial and amyloid pathology. Exploratory multimodal clustering identified three dementia risk profiles—low concern, intermediate, and at risk—which informed an adaptive, tiered detection model. Notably, subjective cognitive concerns assessed by the SCD-Q were strongly associated with overall subjective symptom burden, supporting the use of brief self-report questionnaires as a pragmatic and scalable first-step stratification approach in community-based dementia detection. Community-based, tiered detection pathways for cognitive decline are feasible, acceptable, and meaningful to older adults. Exploratory multimodal profiling suggests that combining subjective, digital, and biological measures may support individualized assessment strategies. However, limited GP engagement highlights the need for stronger integration with primary care to ensure downstream diagnostics and prevention pathways. The proposed framework remains hypothesis-generating and requires prospective validation.

**Clinical trials registered**

CogScreen has been registered at clinical trials (NCT06191952, 2023–12-20).

**Supplementary Information:**

The online version contains supplementary material available at 10.1038/s41598-026-61868-x.

## Introduction

Dementia has emerged as one of the most significant health challenges of the twenty-first century^1^. Because its long prodromal course offers opportunities for prevention, early and timely recognition of cognitive change is a key target of modern preventive medicine^1,2^. Despite growing awareness, early cognitive decline remains largely undetected in primary care and is a systemic challenge even in healthcare systems with standardized access^3,4^. In the U.S. Medicare population, only ~ 8% of expected mild cognitive impairment (MCI) cases are diagnosed^5^, and epidemiological reports from WHO and Alzheimer’s Disease International indicate that around 50% of people with manifest dementia remain undiagnosed even in high-income countries^5–7^. Although general practitioners (GPs) usually recognize cognitive decline, multiple well-documented barriers—including limited consultation time, reimbursement constraints, diagnostic uncertainty in early or subtle presentations, reluctance to label a stigmatized condition, assumptions of ‘normal ageing’, and restricted access to specialist services—often prevent them from initiating further diagnostic steps or referrals^8–10^. In addition, implementation of community-based detection pathways requires effective integration with primary care, including clear referral structures, actionable follow-up procedures, and increasing familiarity with emerging diagnostic tools such as blood-based biomarkers. Public understanding and characterization of cognitive health in the general population remain limited, and little is known about the acceptability of detection approaches outside clinical or specialty settings^11^. Evidence from the Davos Alzheimer’s Collaborative Early Detection Program shows that digital cognitive assessments and blood-based biomarkers can be successfully integrated into routine primary-care workflows, with high completion rates for digital tests (90%) and moderate uptake of blood biomarkers (42%). Importantly, these tools informed subsequent clinical decisions, with more than one-third of eligible patients receiving follow-up investigations such as laboratory testing, structural imaging or specialist referral^12^. Community-based detection therefore represents a complementary strategy, reaching individuals before clinical consultation and those who may not routinely access healthcare services, and enabling low-threshold access to cognitive assessment within real-world care pathways, even though effects on long-term clinical outcomes remain to be established. The CogScreen I study was originally designed to establish a pathway from community-based detection to diagnostic clarification in primary care. However, establishing this connection in practice proved challenging, as GPs often lacked the time, resources, or established procedures to proactively engage with individuals identified outside routine care, highlighting the need for new models that actively integrate primary care into dementia prevention strategies. Consequently, the present analysis focuses on the feasibility and acceptability of multimodal community-based detection approaches combining subjective, digital, and biological measures in non-medical settings. As well as testing feasibility and acceptability, the study sought to explore how these modalities could be combined within a tiered detection pathway that balances assessment depth with individual characteristics and system-level feasibility^10,13–16^.

## Methods

The CogScreen I study investigated whether low-threshold, community-based detection can serve as a pathway for earlier identification of cognitive impairment, by engaging individuals who have not yet sought clinical evaluation and by enhancing public awareness of cognitive health. CogScreen I was a cluster-randomized trial (03/2023–03/2024) conducted in community settings in Munich. The study was approved by the Ethics Committee of the Medical Faculty, LMU Munich (Project No. 22–0786) and registered at ClinicalTrials.gov (NCT06191952, 2023–12-20); written informed consent was obtained from all participants. All methods were performed in accordance with the relevant guidelines and regulations. Eligible participants were older adults (> 60 years of age) with sufficient German language skills, without a preexisting diagnosis of dementia, and with the capacity to provide informed consent and currently receiving routine medical care from a general practitioner (GP). Recruitment occurred at 16 municipal senior centers and one dementia consultation service of Johanniter-Unfall-Hilfe (JUH). Individuals were informed in advance that the events would address dementia and dementia prevention and that a voluntary memory test as part of a study would be offered afterward. Attendees who expressed interest following the informational sessions (or during the dementia consultation) were invited to participate and were screened for eligibility. A study physician provided on-site information and obtained written consent. Sites were randomized to one of three arms:

- A: Subjective Cognitive Decline Questionnaire (SCD-Q) only
- B: SCD-Q + digital cognitive testing (Cognigram)
- C: SCD-Q + digital cognitive testing + blood biomarkers

The nested study design was chosen to evaluate the incremental contribution of progressively more resource-intensive assessment modalities. The SCD-Q served as a universal entry point because it represents a low-cost, scalable, and easily implementable community-based assessment. Digital cognitive testing and blood biomarkers were added in a stepwise manner to explore their potential additional value for multimodal risk profiling and the development of adaptive detection pathways.

### Self-report questionnaire

The SCD-Q consists of 3 introductory questions on metacognitive concern and 24 yes/no items assessing perceived decline across memory (11 items), language (6 items) and executive functions (7 items) over the previous two years. Only the MyCog version was used in this study, as most participants attended the study without an accompanying informant^17^. It has also shown good psychometric properties in community and mixed-age samples, supporting its use for early identification of older adults with SCD who are at increased risk of progression to MCI or dementia^18^. As there is no validated cut-off score, no predefined threshold was applied to classify subjective cognitive decline^17^. Questionnaires were administered on paper, and total scores (0–24) were computed^17^. The questionnaire was administered in paper-based form by trained members of the research team, who assisted participants as needed^17^. To examine how general metacognitive concerns relate to the extent of subjective cognitive complaints, participants were grouped according to the number of affirmative responses (0, 1, 2, or 3 ‘yes’ answers) on the three introductory metacognitive questions (“Do you perceive memory or cognitive difficulties?”, “Would you ask a doctor about these difficulties?”, “Has your memory or cognition declined in the last two years?”) . ANOVA was conducted to test for differences in the SCD-Q total score (items 1–24) across these four response groups. The three introductory SCD-Q items were recorded for exploratory purposes only.

### Digital cognitive testing

Cognigram (Cogstate) was chosen as a brief, examiner-independent digital battery that has been validated in older adults across the cognitive spectrum, including community-dwelling cohorts and primary-care or health-disparity settings showing adequate diagnostic accuracy for MCI and prodromal AD^19–21^. The battery comprises four tasks: Detection (psychomotor speed), Identification (attention), One Card Learning (visual learning/recognition memory), and One Back (working memory). For speed-based tasks, primary outcomes were log-transformed reaction times (seconds). For accuracy-based tasks, primary outcomes were arcsine-transformed proportions of correct responses to reduce skewness and floor/ceiling effects. All outcomes were converted to age-adjusted T-scores (mean = 100, SD = 10) using Cogstate’s normative dataset. Two composite indices were derived: PsyAttStdScr (psychomotor speed/attention; Detection and Identification) and LearnWMStdScr (learning/working memory; One Card Learning and One Back), with higher scores indicating better performance. For clinical interpretation, T-scores were additionally categorized as normal (≥ 90), borderline (80–89), or impaired (< 80), and these categories were communicated to participating general practitioners^19–21^. The digital cognitive assessments were administered in small groups of approximately 4–6 participants and supervised by at least two trained research staff who delivered standardized instructions and a brief demonstration. To accommodate individuals with limited digital experience, staff assisted only with technical issues (e.g., connectivity or device problems), while all cognitive tasks were completed independently without guidance on task content or responses.

### Blood-based biomarkers

The blood-based biomarker panel (plasma amyloid-β1-42/1–40 ratio [Aβ1-42/1–40], phosphorylated tau 181 [pTau181], apolipoprotein E4 [ApoE4], glial fibrillary acidic protein [GFAP], and neurofilament light chain [NfL]) was selected to map onto amyloid (Aβ1-42/1–40), tau (pTau181), and neurodegeneration (NfL) within the AT(N) framework, while additionally capturing astroglial activation (GFAP) and genetic susceptibility (ApoE4)^22–28^. In that study, optimal thresholds for discriminating Alzheimer’s disease from controls were estimated by ROC analysis, yielding cut-offs of pTau181 ≥ 0.352 pg/mL, Aβ1–42/1–40 ratio ≤ 0.009, ApoE (plasma) ≥ 3.616 µg/mL, and NfL ≥ 1.604 pg/mL^28^. GFAP was retained as a continuous marker because no robust diagnostic cut-point could be established. Samples were stored at –80 °C until analysis and processed in a single batch to minimize assay variability. All assays were performed with reagents and consumables supplied by Roche Diagnostics and were designated for research use only; assays were measured using the cobas e 402 platform (Roche Diagnostics).

### Report to physicians

Results from the SCD-Q and Cognigram assessments were summarized into standardized feedback letters addressed to participants’ general practitioners (GPs). GPs were not informed in advance that their patients had participated in the study; they were notified through the feedback letter sent after completion of the assessment. The reports included composite scores and the overall classification, while explicitly emphasizing the exploratory nature of the findings. Physicians were advised that the results may indicate an increased likelihood of cognitive impairment (e.g., mild cognitive impairment or early Alzheimer’s disease) but do not replace a comprehensive neuropsychological evaluation. Decisions regarding further diagnostic work-up, including referral to specialized memory clinics, remained at the discretion of the GP. No formal training regarding blood-based biomarkers or digital cognitive assessments was provided to participating GPs as part of the study protocol. Furthermore, no standardized referral pathway or reimbursement framework for community-based cognitive screening was available during the study period.

### Feedback from general practitioners and participants

A subsample of 25 participants and 10 general practitioners was interviewed using a structured format to capture experiences with recruitment, testing, and result communication^29,30^. Participant interviews were drawn through stratified random sampling from the CogScreen database and conducted in person at the Department of Psychiatry and Psychotherapy, LMU Hospital. Recruitment continued until data saturation was reached. Interviews followed a structured guide addressing motivations for participation, experiences with recruitment and testing (including digital assessments), perceptions of result communication, and suggestions for procedural improvement. Qualitative analysis used a thematic content approach. Transcripts were coded in MAXQDA using deductive categories derived from the interview guide and inductive codes for emerging themes. To ensure analytic rigor, 5–10% of interviews were double-coded, and discrepancies resolved by consensus. Codes were iteratively refined and organized into higher-order themes capturing feasibility, acceptability, and barriers to implementation. Only methodological aspects relevant to feasibility are reported here; full qualitative results are presented in a separate manuscript^29,30^.

### Speech analysis

A subsample of 68 participants completed an optional exploratory digital speech task (ki:e SB-C)^31,32^. Only individuals who had completed the full CogScreen assessment were invited. Uptake was limited, as several older adults were unfamiliar with or distrustful of an automated telephone system and preferred not to provide spoken responses to a voicebot. Consequently, the speech subsample reflects willing participants rather than the full study cohort. The ki:e SB-C protocol was delivered via automated telephone call and included a 60-s semantic verbal fluency task (“animals”) and four immediate recall trials from the Rey Auditory Verbal Learning Test (RAVLT). Audio recordings were processed through ki:e’s automated pipeline, extracting semantic (e.g., clustering, switching), temporal (latencies, transition times), lexical, and prosodic features^31,32^. The speech component served as an exploratory feasibility assessment, examining acceptability, completion rates, and missingness patterns, and providing preliminary correlations with CogScreen outcomes. Speech-derived variables were not included in the main statistical models.

### Statistical analyses

According to the preregistered protocol, GP-verified diagnostic outcomes and subsequent memory-clinic evaluations were originally planned as external validation outcomes. Because downstream GP engagement and referral activity were substantially lower than anticipated, these outcomes could not be evaluated as planned and are therefore not reported in the present manuscript.

### Primary endpoints: Feasibility and acceptability

Analyses focused on descriptive and comparative summaries of participation rates, completion rates, and patterns of missingness across study arms. Quantitative feasibility indicators (e.g., proportion completing SCD-Q, digital testing, biomarkers) were compared using ANOVA or Kruskal–Wallis tests. Acceptability was evaluated through structured questionnaire items and categorical summaries of participant and GP responses. Qualitative interview data (reported in a separate manuscript) informed contextual interpretation but were not entered into statistical models.

### Secondary exploratory endpoints: Cognitive structure, multimodal profiles, and biomarker associations

All secondary analyses were restricted to numeric variables, with placeholder values coded as missing. Variables with near-zero variance or excessive collinearity were excluded, and all retained measures were z-standardized.

### Exploratory factor analysis (EFA)

EFA (maximum-likelihood extraction, oblimin rotation) was applied to age-adjusted Cognigram T-scores to examine latent cognitive dimensions in the pooled sample. Models were adjusted for sex and education. Suitability was assessed using the KMO index and Bartlett’s test of sphericity.

### Exploratory cluster analysis

K-means clustering (k = 2–6) was performed on z-standardized composite scores (PsyAttStdScr; LearnWMStdScr) after removing multivariate outliers (Mahalanobis distance, p < 0.001). The optimal solution was determined by silhouette width and the gap statistic. The aim of the clustering was to explore whether distinct cognitive profiles could be identified that may serve as a basis for future risk profiling or early detection models.

### Correlation analysis

Associations across modalities—subjective SCD-Q scores, objective cognitive performance, and plasma biomarkers—were examined using partial correlations controlling for age, sex, and education. Pearson or Spearman coefficients were reported depending on variable distributions, with 95% CIs derived using Fisher’s z transformation. Multiple testing was controlled using the false discovery rate (Benjamini–Hochberg).

All analyses were conducted in RStudio (version 2025.05.1). Variable definitions and non-missing counts are listed in Supplement Table S1.

## Results

Enrollment was completed swiftly between March 2023 and March 2024, ahead of the originally projected 1.5 years.

### Recruitment and demographics

In total, 16 senior centers and 1 dementia consultation hour at the Johanniter-Unfall-Hilfe (JUH) participated. A total of 473 participants aged ≥ 60 years were recruited, 441 (95.0%) completed the study (15 withdrew consent, 8 discontinued testing).

**Fig. 1:**
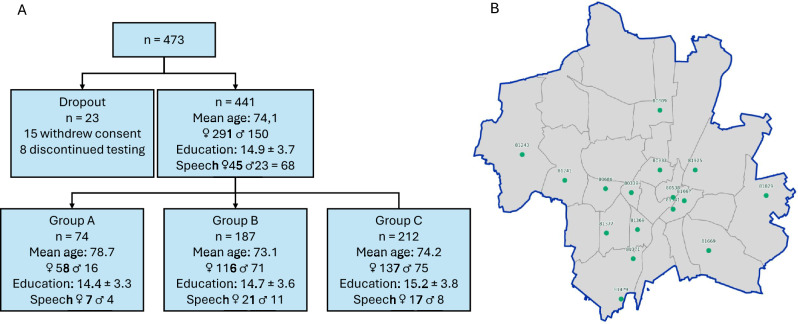
**A** Study design and intervention groups. Group A: SCD-Q only; Group B: SCD-Q + digital cognitive testing (Cognigram); Group C: SCD-Q + digital testing + blood biomarkers (Aβ, pTau181, GFAP, NfL). A total of 473 participants were recruited; 441 completed all study procedures. **B** Geographic distribution of the 17 recruitment sites (16 senior service centers and one JUH dementia consultation service) in the greater Munich area. Data source administrative boundaries of Munich districts © City of Munich, licensed under Data license Germany – attribution – Version 2.0 (dl-de/by-2–0), www.govdata.de/dl-de/by-2-0. Dataset adapted for visualization.

**Fig. 2:**
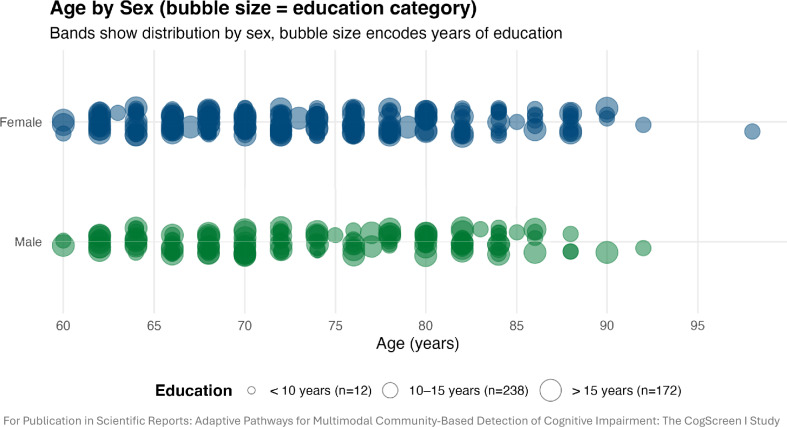
Age distribution by sex (bubble size = education category).

Participants were assigned to Group A (SCD-Q only, n = 74), Group B (SCD-Q plus digital testing, n = 187), or Group C (SCD-Q plus digital testing plus blood biomarkers, n = 212). Recruitment and participant flow are shown in Fig. 1. Participation was lowest in Group A (88.2%) compared with Groups B (95.3%) and C (96.5%). This pattern is consistent with qualitative feedback indicating that the questionnaire-only format was perceived as the least informative and offered limited personal benefit, whereas digital cognitive testing—and especially the biomarker assessment—were viewed as more meaningful, medically relevant, and worth the effort. As a result, the more comprehensive the assessment modality, the higher the participation rate observed in Groups B and C. The mean age across the sample was 74.1 years (SD = 7.6, range 60–95); 66% were women and 61% reported tertiary education (≥ 13 years). Group A participants were significantly older (78.7 years) and less often male (21%) than those in Groups B (73.1 years, 38% male) and C (74.2 years, 35% male). (Table 1, Fig. 1 and 2).

**Table 1:** Baseline characteristics by study arm (A: SCD-Q only; B: SCD-Q + Digital; C: SCD-Q + Digital + BBBM).

Characteristic	A	B	C	Total	p
Total (N = 473)	n = 74	n = 187	n = 212		
Age, years (mean ± SD)	78.7 ± 7.5	73.1 ± 7.2	74.2 ± 7.3	74.1 ± 7.6	< 0.001
Education, years (mean ± SD)	14.4 ± 3.3	14.7 ± 3.6	15.2 ± 3.8	14.9 ± 3.7	0.329
**Education categories**					
Education (n): Primary (≤ 9)	1	7	5	13 (3%)	
Education (n): Secondary (10–12)	20	48	49	117 (25%)	
Education (n): Tertiary (≥ 13)	40	110	140	290 (61%)	
Education: missing				53 (11%)	
Sex distribution					0.169
Sex: Female	58 (78%)	116 (62%)	137 (65%)	311 (66%)	
Sex: Male	16 (22%)	71 (38%)	75 (35%)	162 (34%)	

Baseline demographic characteristics by study arm. Continuous variables are presented as mean ± SD and compared using ANOVA; categorical variables are shown as n (%) and compared using Kruskal–Wallis tests. Percentages are based on available data.

### Self-report (SCD-Q)

**Fig. 3:**
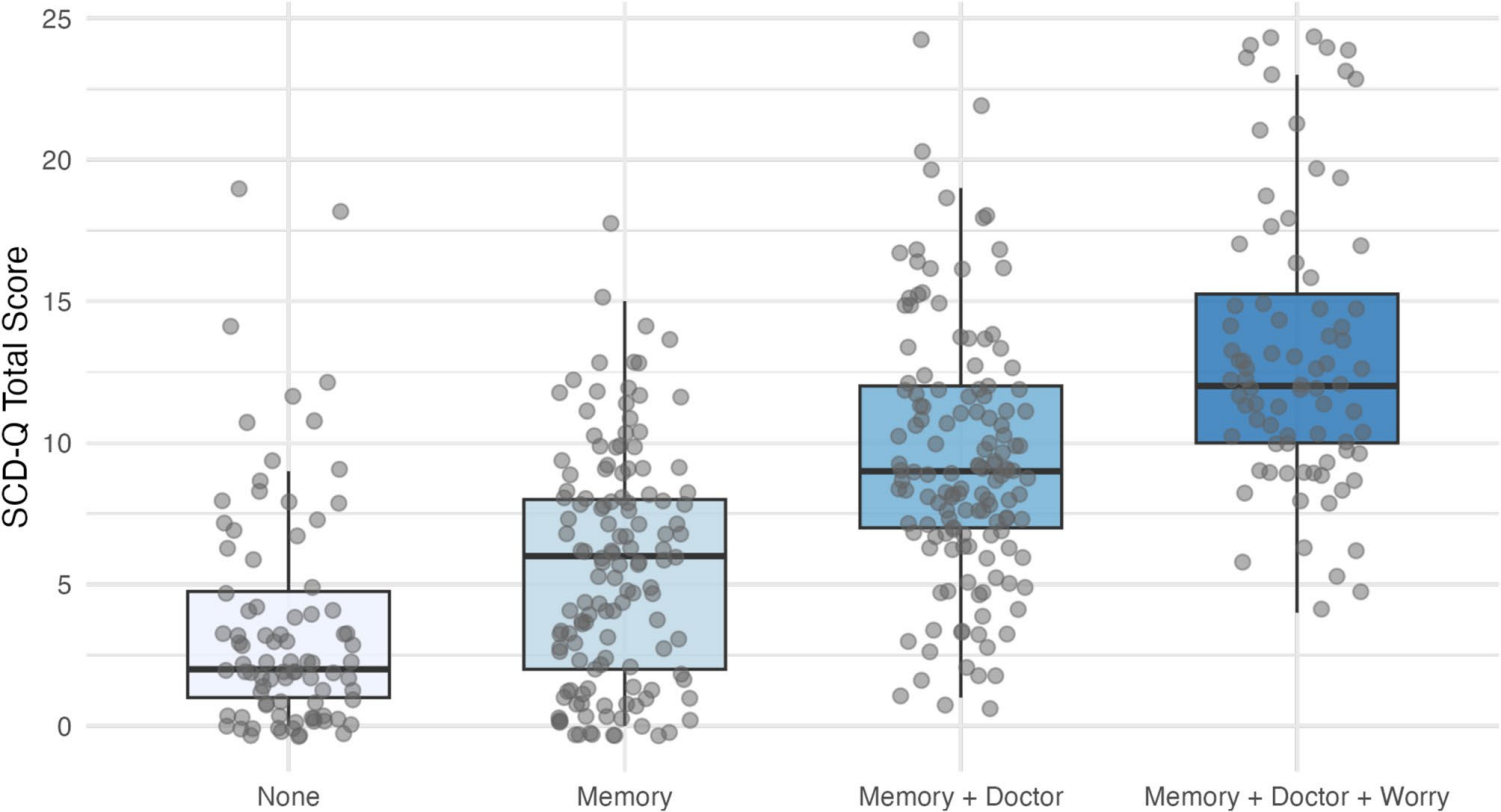
Association between endorsement of introductory detection questions and SCD-Q total score. Participants with more “yes” responses achieved higher SCD-Q total scores. ANOVA confirmed group differences (F(3, 432) = 83.7, p < .001); Tukey tests showed significant pairwise differences between all groups (all p ≤ .006). Introductory questions: 1) Do you notice memory difficulties? 2) Would you seek medical advice for these concerns? 3) Do these difficulties cause you worry?.

The SCD-Q results underline that subjective cognitive complaints represent a meaningful and structured signal in community-based detection. Participants’ endorsement of the three introductory detection items (“concern,” “perceived decline,” “medical consultation”) showed a strong graded association with the SCD-Q score: individuals reporting none, one, two, or all three concerns had mean total scores of 3.5, 5.6, 9.4, and 13.3, respectively (Table 2). This monotonic increase was demonstrated by one-way ANOVA (F(3, 432) = 91.24, p < 0.001, η^2^ = 0.37), indicating a large effect on the number of endorsed intro questions on SCD-Q total score. Exploratory factor analysis supported a multifactorial rather than unitary structure of the SCD-Q, with clusters predominantly reflecting memory-related, language-related, and executive-attentional difficulties. Several cross-loadings were present, indicating that real-world subjective complaints rarely fall into discrete categories but instead reflect overlapping functional challenges. This heterogeneity demonstrates that the SCD-Q captures metacognitive awareness of cognitive change rather than a single, global dimension of decline. Importantly, individuals endorsing ≥ 2 introductory questions showed higher levels of subjective cognitive concerns compared to those with fewer endorsements, reinforcing the potential value of these brief items for early-stage case finding, even though no established cut-off for subjective cognitive decline exists. Together, these findings show that a condensed version of the SCD-Q — focusing on the most informative introductory and domain-specific items — could enhance efficiency in both research and clinical community settings (see Tables 2 and 3, Fig. 3, and Table S2).

**Table 2:** SCD-Q total score stratified by number of positive intro questions (0–3).

Intro yes	N	Mean	SD	Min	Max
0	97	3.535	4.078	0	19
1	143	5.565	4.133	0	18
2	148	9.367	4.492	1	24
3	85	13.263	5.145	4	24
**Total score**					
SCD-Q1 to Q24	436	7.789	5.562	0	24
ANOVA (F(3, 432) = 91.24, p < 0.001, η^2^ = 0.37)

**Table 3:** Key factor analysis results of self-report questionnaire (SCD-Q).

Factor / Domain	Item(s) (scdq)	Factor Loading	Communality
Memory Complaints	9, 10, 11	.62 – .78	.41 – .58
Language Difficulties	12, 13, 14, 17	.55 – .81	.39 – .61
Executive Function	16, 20, 21, 22	.44 – .70	.32 – .55
Attention / Concentration	1, 2, 3, 7	.42 – .68	.30 – .50
Daily Functioning / Mixed	4, 5, 6, 15, 18, 19, 23, 24	.40 – .66	.28 – .52

Only salient loadings (≥ .40) are shown. Factor loadings and communalities derived from maximum-likelihood EFA with oblimin rotation.

### Digital cognitive testing

A digital cognitive assessment using the Cognigram battery provided standardized indices of accuracy, reaction time, and age-adjusted composite T-scores (Table S3). Accuracy was high for the speeded tasks (Detection: 97.5%, SD = 4.8%; Identification: 97.0%, SD = 5.2%), with corresponding arcsine-transformed values of 1.48 (SD = 0.13) and 1.47 (SD = 0.14). Reaction times showed the expected task-dependent gradients, ranging from 606 ms (SD = 288) for Detection to 1,808 ms (SD = 690) for One-Card Learning. Accuracy in the more demanding One-Back task was lower (90.8%, SD = 10.9%), consistent with its higher cognitive load. Detailed descriptive statistics are provided in Table S3. Exploratory factor analysis (maximum-likelihood extraction, oblimin rotation) identified two latent cognitive factors that together explained 60.3% of total variance (Table 4, Fig. S3). A ‘psychomotor speed and attention’ factor (PsyAtt) was defined by the Detection and Identification tasks, while a ‘learning and working memory’ factor (LearnWM) emerged from One-Card Learning and One-Back. Moderate inter-factor correlations (r = 0.25–0.42) indicated related but separable domains. Although the overall KMO value was low (KMO = 0.50), reflecting limited shared variance across heterogeneous speed- and accuracy-based tasks, Bartlett’s test of sphericity was highly significant (χ^2^(105) = 14,699.82, p < 2 × 10⁻^1^⁶), supporting factorability. Composite T-scores derived from these factors showed mean values of 97.6 (SD = 9.8) for PsyAttStdScr and 96.2 (SD = 10.1) for LearnWMStdScr. Age and education contributed significantly to performance variance, with higher education partly mitigating age-related decline. Taken together, the Cognigram battery captured two core, psychometrically distinct domains of objective cognition—attentional control and memory processing—that complement subjective self-report and support scalable detection in community and non-specialist settings (Table 4; Table S3; Fig. S2–S3).

**Table 4:** Key faktor analysis results: digital cognitive battery.

Cognitive domain	Factor loading	Communality
**Working memory & attention**		
One-Back Accuracy (T-score)	0.99	0.032
One-Back Accuracy (raw)	0.945	0.113
One-Back Working Memory (T-score)	0.69	0.468
Identification Accuracy	1.002	0.002
**Learning & memory**		
One-Card Learning Accuracy (T-score)	1.002	0.003
One-Card Learning Accuracy (raw)	1.002	0.004
One-Card Learning Memory (T-score)	0.627	0.488
**Psychomotor speed & reaction time**		
Detection Reaction Time (raw)	-0.823	0.283
Detection Accuracy	0.954	0.09
Detection Working Memory (T-score)		0.907
One-Back Reaction Time (raw)	-0.521	0.563
**Cognitive control & processing time**		
Identification Reaction Time (raw)	0.942	0.119
Identification Attention (T-score)	-0.85	0.221
One-Card Learning Attention (T-score)	0.628	0.617

### Blood biomarkers

Plasma biomarker assays were available for 220 participants (Table 5). Mean concentrations were: Aβ1-40 = 0.292 ng/mL (SD = 0.052), Aβ1-42 = 33.7 pg/mL (SD = 8.4), Aβ1-42/40 ratio = 0.117 (SD = 0.036), pTau181 = 0.841 pg/mL (SD = 0.332), GFAP = 0.081 ng/mL (SD = 0.044), and NfL = 1.97 pg/mL (SD = 1.03). Distributions showed substantial interindividual variability, with upper values reaching 8.5 pg/mL for NfL and 0.406 ng/mL for GFAP. ApoE4 concentrations (mean = 3.38 mg/mL, SD = 6.79) showed minimal communality and did not contribute meaningfully to the latent structure. Compared with established diagnostic thresholds, biomarker patterns were generally consistent with low clinical pathology in this community sample. The Aβ1–42/1–40 ratio was markedly higher than clinical cut-points (mean 0.117 vs. ≤ 0.009), indicating an amyloid-normal profile for nearly all participants. Absolute Aβ1-42 and Aβ1-40 values also aligned with non-clinical reference ranges. By contrast, pTau181 and NfL exhibited mean levels above the ROC-derived thresholds (0.841 pg/mL and 1.97 pg/mL, respectively), while GFAP displayed a low mean alongside a substantial pathological range (exceeding 0.25 ng/mL in certain individuals).

**Table 5:** Blood-based biomarkers.

BBBM	N	Mean	SD	Min	Max	Communality	Uniqueness
Aβ1-40 (ng/ml)	220	0.292	0.052	0.004	0.476	0.995	0.005
Aβ1-42 (pg/ml)	220	33.711	8.368	2.090	55.500	0.995	0.005
Aβ1-42/1–40 ratio	220	0.117	0.036	0.028	0.565	0.407	0.593
Apoe4 (mg/ml)	220	3.384	6.787	0.041	37.5	0.011	0.989
Gfap (ng/ml)	220	0.081	0.044	0.014	0.406	0.769	0.231
Nfl (pg/ml)	220	1.966	1.027	0.572	8.5	0.478	0.522
ptau181 (pg/ml)	220	0.841	0.332	0.300	2.45	0.350	0.650

Exploratory factor analysis (maximum-likelihood extraction, oblimin rotation) confirmed the suitability of the biomarker matrix (KMO = 0.63, Bartlett’s χ^2^(10) = 376.86, p < 7.9 × 10⁻⁷^5^; Fig. S4). A two-factor solution explained 64% of the variance. The first factor reflected neurodegeneration/glial injury, defined by GFAP (loading 0.88), NfL (0.69), and pTau181 (0.56). The second captured amyloid biology, with strong positive loadings for Aβ1-40 and Aβ1-42 and a negative loading for the Aβ1-42/1–40 ratio, consistent with known physiological relationships. Communalities were high (e.g., Aβ1-42 h^2^ = 0.995, GFAP h^2^ = 0.769), indicating robust shared variance.

Biomarkers showed functional relevance: GFAP correlated with attentional performance (β = –0.21, p = 0.01), and pTau181 showed a trend-level association with working memory (β ≈ 0.15). Together, these findings delineate two complementary biological axes—neurodegeneration/glial activation and amyloid status—that integrate meaningfully with digital and self-report measures to characterize early multimodal signatures of cognitive vulnerability (Table 5, Fig. S3).

### Clustering analyses

**Fig. 4:**
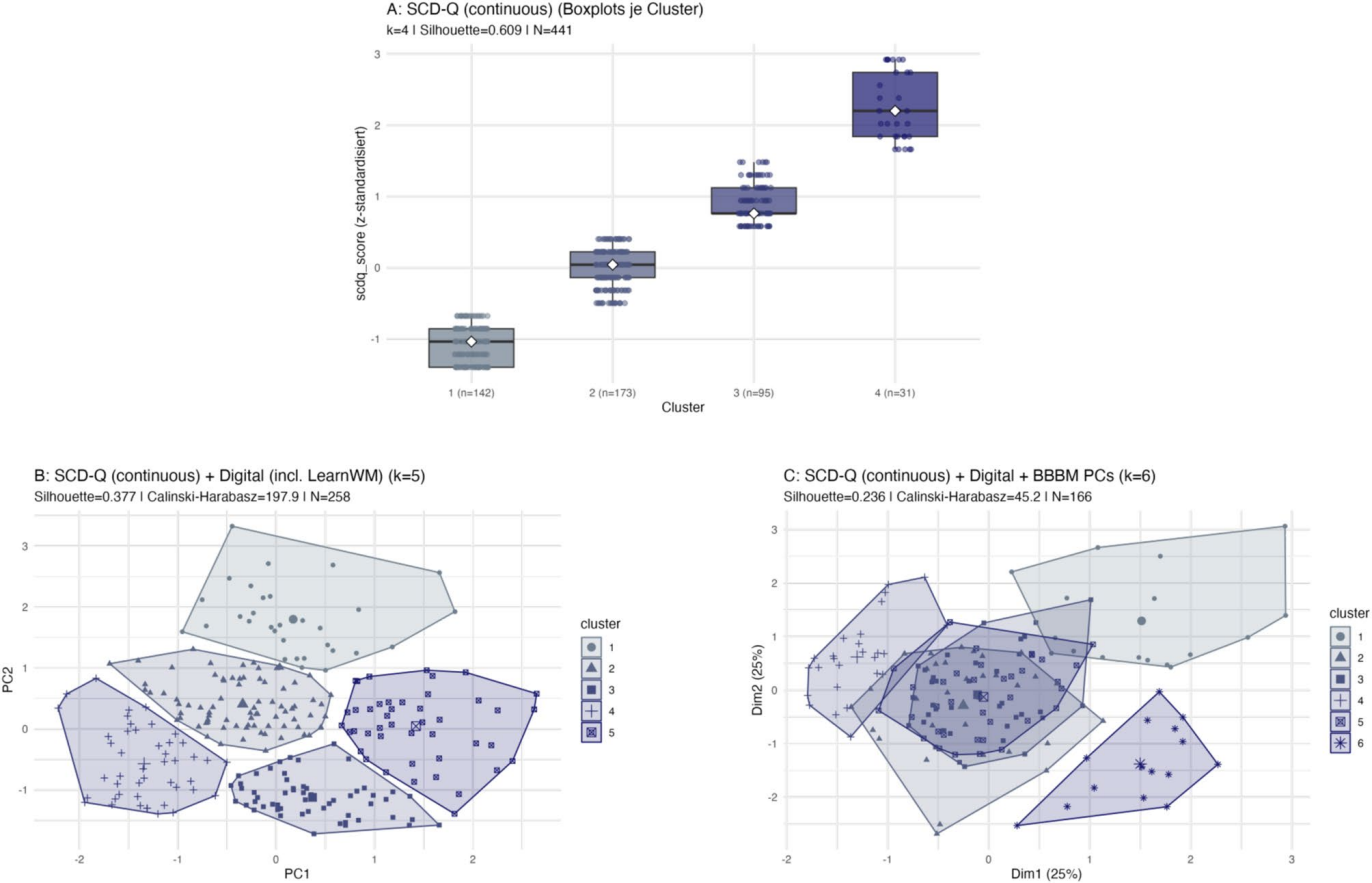
Cluster solutions derived from (A) SCD-Q, (B) SCD-Q + digital tests, and (C) multimodal data (SCD-Q, digital tests, biomarkers). Clusters identified by k-means with silhouette-based k selection. Boxplots shown for (A); PCA plots with cluster membership (color-coded) for (B–C). BBBM principal components: PC1 = glial/axonal injury (GFAP, NfL, pTau181), PC2 = amyloid (Aβ42/40 ratio). See Table 7 for interpretation. Interpretation: (A) Clusters represent different levels of subjective complaints (low → high SCD-Q). No additional dimensions included. (B) Clusters split into groups with (i) low complaints + normal digital performance, (ii) high complaints + reduced attention/working memory, and mixed intermediate groups.(C) Clusters represent combinations of complaint severity, digital test deficits, and biomarker abnormalities: (a) low SCD-Q + normal digital + normal BBBM, (b) high SCD-Q + impaired attention, (c) pathological BBBM profile (low Aβ ratio, elevated GFAP/NfL/pTau181).

To examine whether subjective, cognitive, and biological measures form distinct participant profiles, k-means clustering was applied across three progressively enriched models (Fig. 4, Table 6). Although overall cluster compactness decreased from Model A to C (silhouette: 0.61 → 0.38 → 0.24), the interpretability and discriminative value of the clusters increased markedly. In Model A (SCD-Q only), four clusters emerged (n = 31–173), almost entirely driven by subjective cognitive complaints (η^2^ = 0.92). Mean SCD-Q values ranged from z = –1.1 (minimal concerns) to z =  + 1.9 (pronounced perceived decline), indicating that self-report alone primarily captured a graded continuum of subjective symptom severity. Adding digital cognitive testing in Model B (SCD-Q + digital) yielded five clusters (n = 31–80). While subjective report continued to contribute strongly (η^2^ = 0.78), digital performance added substantial differentiation, particularly in learning/working memory (η^2^ = 0.45) and psychomotor attention (η^2^ = 0.41). Cluster means ranged from high-performing groups (PsyAtt z ≈ + 0.6, LearnWM z ≈ + 0.4) to groups with notable slowing or memory inefficiency (PsyAtt z ≈ –1.0, LearnWM z ≈ –0.9), even when subjective concerns were modest. The fully multimodal Model C (SCD-Q + digital + biomarkers) produced six clusters (n = 15–39) jointly shaped by cognitive and biological variation. Psychomotor attention (η^2^ = 0.68), the amyloid Aβ1–42/1–40 ratio (η^2^ = 0.53), and glial/axonal markers including GFAP, NfL, and pTau181 (η^2^ = 0.51) contributed strongly to subgroup separation. Biomarkers exhibited wide gradients across clusters—for example, GFAP ranged from z = –0.8 to + 2.1, NfL from –0.5 to + 1.7, pTau181 from –0.3 to + 1.4, and the Aβ1–42/1–40 ratio from + 0.4 to –0.9—revealing biological differences not detectable in Models A or B.

**Table 6:** Comparison of cluster characteristics across models.

Model	Variables included	Optimal k	N per cluster	Silhouette	Main discriminative features
**A** SCD-Q	SCD-Q score	4	C1: 95; C2: 31; C3: 173; C4: 142	0.609	SCD-Q (η^2^ = 0.917)
**B** SCD-Q + Digital	SCD-Q score, PsyAtt, LearnWM	5	C1: 31; C2: 80; C3: 57; C4: 46; C5: 44	0.377	SCD-Q (η^2^ = 0.776) LearnWM (η^2^ = 0.450) PsyAtt (η^2^ = 0.407)
**C** SCD-Q + Digital + BBBM	SCD-Q score PsyAtt LearnWM, BBBM PCs	6	C1: 17; C2: 33; C3: 39; C4: 26; C5: 36; C6: 15	0.236	PsyAtt (η^2^ = 0.677) BBBM-PC2 (η^2^ = 0.533, driven by Aβ ratio) BBBM-PC1 (η^2^ = 0.506, GFAP/NfL/pTau) SCD-Q (η^2^ = 0.470)

Factor loadings represent the highest loading per variable in the seven-factor solution of speech-derived measures (Semantic Verbal Fluency, ‘animals’). Communalities (h^2^) indicate the proportion of variance explained by the extracted factors. Values < 0.40 suggest limited representation of the variable in the latent factor structure.

Although Model C generated six numeric clusters, these converged into three coherent meta-profiles. A compensated/low-risk profile showed near-average SCD-Q scores (z ≈ 0), preserved digital performance (PsyAtt z ≈ + 0.3; LearnWM z ≈ + 0.2), and biomarker levels in the normal range. A subjective-dominant profile exhibited elevated SCD-Q (z ≈ + 1.2) despite normal cognitive and biomarker outcomes, consistent with heightened concern without measurable impairment. A biologically high-risk profile showed both reduced cognitive performance (PsyAtt z ≈ –0.7; LearnWM z ≈ –0.8) and abnormal biomarkers (GFAP/NfL/pTau181 >  + 1; Aβ1–42/1–40 ratio < 0), consistent with emerging neurodegenerative processes.

Overall, the multimodal model revealed biologically and cognitively meaningful subgroups that were not identifiable from questionnaires or digital tests alone, underscoring the added value of integrated, multi-domain detection pathways.

### Regression analyses

**Fig. 5:**
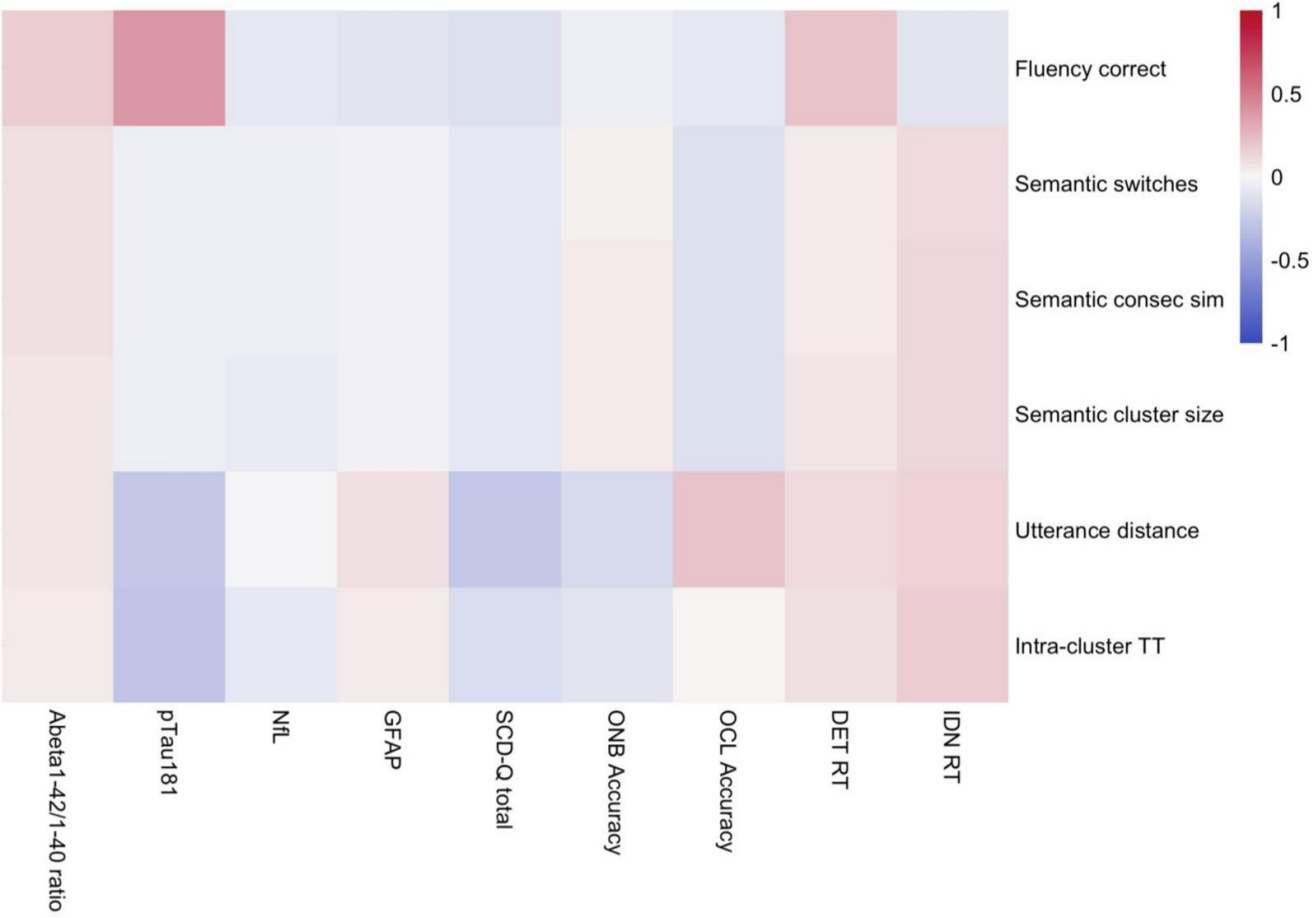
Heatmap of partial correlations between biomarker factors (rows) and cognitive/speech outcomes (columns). Red = positive correlation; blue = negative; white = null. Asterisks denote FDR-corrected significance. Biomarkers: GFAP = astroglial marker; NfL = axonal injury; pTau181 = tau pathology; Aβ42/40 = amyloid pathology. Cognitive composites: PsyAtt T = psychomotor/attention; LearnWM T = learning/working memory; DET RT = detection RT; IDN RT = identification RT; ONB Accuracy = one-back working memory; OCL Accuracy = one-card learning. Speech measures: Fluency correct = number of animals; Semantic cluster size = mean cluster size; Semantic switches = number of switches; Consecutive similarity = semantic relatedness of successive words; Utterance distance = mean distance between utterances; Intra-cluster TT = intra-cluster transition time. Questionnaire: SCD-Q total = Subjective Cognitive Decline Questionnaire score.

Regression analyses identified demographic and biomarker predictors of digital cognitive performance (Fig. 5, Table 7). Age remained a significant predictor of cognitive performance even though the outcomes were age-adjusted composite T-scores. Specifically, higher age was associated with lower scores on the psychomotor speed/attention composite (PsyAttStdScr; β = –0.28) and the learning/working-memory composite (LearnWMStdScr; β = –0.50). This indicates that the normative age-correction applied by Cognigram reduces, but does not fully eliminate, ageing-related variance. Education was a strong positive predictor of learning/working memory (β = 0.73, p < 0.001), highlighting the role of cognitive reserve. Higher blood-based biomarker levels were associated with worse cognitive outcomes. Specifically, elevated GFAP predicted lower attention performance (β = –0.21, p = 0.01; FDR p = 0.05), while higher pTau181 showed a trend-level association with reduced learning/working memory (β = 0.15, p = 0.09). Although including biomarker information improved model fit—raising explained variance from adj. R^2^ = 0.02 to 0.07–0.11 for attention and from 0.15 to 0.22–0.25 for memory—the overall amount of variance accounted for remained modest and indicates other factors not captured by the current models.

**Table 7:** Backward elimination regression results.

Outcome	Predictor	Beta	SE	t	p	Adj. R^2^	n
psyattstdscr	Gfap	-0.069	0.018	-3.861	0.000	0.08234644	156
learnwmstdscr	age, education	-0.608	0.109	-5.575	0.000	0.22214124	153
learnwmstdscr	age, education	0.6431	0.123	3.031	0.003	0.22214124	153

### Speech analysis

Automated analysis of speech-derived variables was available for 66 participants (Table S4). Basic verbal fluency output was comparable to established neuropsychological measures. Participants produced a mean of 20.4 animal names (SD = 5.9) within 60 s, closely matching performance levels reported for cognitively healthy adults in the Consortium to Establish a Registry for Alzheimer’s Disease (CERAD) “Animals” test (approximately 21 words for individuals aged 50–70) and falling within the expected lower-normal range for older adults^33,34^. The number of semantic switches (M = 5.7, SD = 2.0) and mean semantic cluster size (M = 3.4, SD = 1.3) were consistent with ranges observed in established verbal-fluency norm studies, indicating that participants’ clustering and switching behavior aligned with typical patterns reported for older adults^35^. Principal component analysis indicated a multifactorial structure (KMO = 0.56, Bartlett p < 0.001; Fig. S3–S4, Table 8). Seven factors were extracted, reflecting semantic similarity within clusters, overall task performance, clustering versus switching, temporal dynamics, and bin-specific similarity measures. This multidimensionality highlights the richness of automatically derived features, but also complicates interpretation given the large number of interdependent subitems. Correlations with cognitive outcomes and blood-based biomarkers were weak (r ≈ 0.1–0.3) and did not survive FDR correction (Table S5). A trend-level association emerged between higher pTau181 and lower semantic fluency (r = –0.40, p = 0.06, n = 23).

**Table 8:** Key factor analysis results for speech analysis.

Speech tasks	Factor loading	Communality
**Semantic clustering**		
Mean consecutive similarity	0.899	0.65
Mean intracluster similarity	0.782	0.61
Mean consecutive similarity	0.456	0.42
**Task performance**		
Correct count animals	0.823	0.55
**Semantic clustering vs. switching**		
Mean cluster size	0.922	0.68
Switches	-0.872	0.59
Mean intercluster similarity (overall)	0.578	0.44
Mean intracluster similarity (bin 10)	-0.473	0.37
**Temporal dynamics**		
Mean utterance distance (animals)	0.807	0.56
Mean intracluster transition time (intra)	0.662	0.48
Mean intercluster transition time (inter)	0.475	0.39
Switches	-0.486	0.36
**Bin-specific similarity**		
Mean consecutive similarity (bin2)	0.582	0.41
Mean consecutive similarity (bin5)	0.454	0.33
**Temporal clustering**		
Mean cluster size	0.728	0.52
Mean consecutive similarity (bin1)	-0.647	0.43

Backward selection was performed using BIC (stepAIC with k = log(n)). Predictors were entered in three blocks (Block 1: age, sex, education; Block 2: SCD-Q; Block 3: blood biomarkers). Variables not retained in the final model are listed under “removed”. Results show predictors retained, predictors removed, regression coefficients (Beta, SE, t, p), BH–FDR correction for blood biomarkers, adjusted R^2^, and sample size (N).

### General practitioner feedback and referral outcomes

Structured feedback was sought from all general practitioners (GPs) named by study participants. Despite multiple contact attempts, responses were often limited, and in several cases, feedback reflected skepticism or perceived interference with existing care routines. Some GPs expressed concerns about the clinical relevance of study findings, particularly regarding the digital cognitive assessments, which were occasionally viewed as experimental rather than actionable tools. This highlights the need for future work to use community engagement methods to co-develop more acceptable and actionable pathways with GPs.

In total, structured feedback was obtained from 25 GPs, and 10 GPs participated in qualitative follow-up interviews, which will be reported in a separate manuscript. Most respondents cited time and resource constraints as major barriers but also emphasized that broader validation, reimbursement mechanisms, and integration with established referral pathways could improve acceptance.

### Textbox 1: Theoretical adaptive community case-finding model

In order to integrate the multimodal findings of CogScreen I conceptually, a theoretical model of adaptive case-finding could be developed. This framework would illustrate how subjective, cognitive and biological indicators could interact to inform approaches to community-based dementia prevention that are both ethically sound and resource-efficient.

#### Entry and ethical rationale

Screening could begin with three introductory questions probing^17^:

- “Do you perceive memory problems?”
- “Would you consult a doctor about your concerns?”
- “Do these problems worry you?”

If two or more of these are endorsed, this would ethically justify further assessment, ensuring that any ‘confrontation’ with possible decline is proportional to the individual’s perceived experience.

#### Psychometric Core measurement domains

- Subjective–metacognitive: a shortened SCD-Q focusing on awareness, language and executive control (items 9–11, 12–14, 16 and 20–22).
- Informant perspective: when a caregiver or family member is available (particularly for individuals aged > 60 years), a brief informant rating (e.g., the TheirCog component of the SCD-Q or AD8) could complement self-report to increase diagnostic accuracy and contextual validity^17,36^.
- Cognitive–functional: digital measures of psychomotor attention (Detection and Identification) and learning/working memory (e.g. One-Card Learning and One-Back).
- Biological: blood biomarkers emphasising GFAP and pTau181, which may provide more stable and disease-relevant signals of neurodegeneration and glial activation than amyloid-based ratios given the instability of plasma Aβ measures^37,38^.

#### Adaptive decision levels

Depending on the combination of these indicators, individuals may fit into one of three empirically derived profiles:

- Compensated/low risk: preserved digital performance and normal biomarkers;
- Subjective-dominant: high concern, but normal objective findings;
- Biological high risk: reduced digital performance and elevated GFAP/pTau181.

#### Age- and education-sensitive adaptation

Procedures should include age-adjusted z-scoring and speed-independent digital tasks to maintain psychometric comparability and reduce cognitive load.

## Discussion

This cluster-randomized trial demonstrates the feasibility and acceptability of conducting multimodal cognitive detection—integrating self-report, digital testing, and blood biomarker collection—in non-medical community settings. Completion rates across all study arms exceeded 85%, indicating strong acceptability among participants. Feasibility was supported by the successful delivery of all procedures on-site by research staff with limited clinical experience, with the full assessment completed within approximately 20 min. Digital testing required minimal technical assistance, and biological sample collection proceeded without major issues, showing that multimodal detection can be effectively implemented outside clinical environments.

In line with our pre-registered analysis plan, the primary endpoints were intended to comprise GP-verified diagnostic outcomes and memory clinic results. These results would serve as external validators for the multimodal detection model. However, despite the exceptionally rapid recruitment reflecting high community engagement, the response rate from participating GPs was much lower than anticipated. Few participants were referred for clinical evaluation, and most GP feedback forms were not returned. Consequently, the planned primary and secondary outcome analyses, particularly those linking community-based detection results to formal diagnostic endpoints, could not be undertaken. This substantially limits our ability to draw diagnostic or predictive inferences from the data, meaning that the current findings should be interpreted as focusing on feasibility and patterns rather than providing diagnostic confirmation. A separate qualitative follow-up study is underway to analyze the barriers reported by GPs, organizational constraints and perceptions of community detection. This study will be reported in a separate manuscript^30^.

### Principal findings

The three domains assessed in CogScreen I—subjective experience, digital cognitive performance, and biological markers—contributed distinct but complementary information. The SCD-Q reflected heterogeneous self-perceptions of cognitive change, with introductory items (e.g., “memory problems”, “would consult a doctor”, “worry”) showing strong graded associations with total scores, supporting its metacognitive nature^39^. Importantly, the strong graded relationship between the introductory SCD-Q items and the overall burden of subjective cognitive complaints suggests that brief self-report questionnaires may serve as a pragmatic first-step stratification tool in community-based detection pathways, allowing more resource-intensive assessments to be targeted to individuals with higher levels of concern.

Digital composites for psychomotor attention and working memory were age-adjusted, yet age remained a significant predictor of poorer performance. This suggests that normative correction does not fully compensate for ageing-related variability. Digital proficiency likely also influences performance in older adults but could not be assessed in this exploratory study^40^.

Biomarker analyses revealed two separable axes—amyloid pathology and neurodegeneration/glial injury—with GFAP emerging as the strongest correlate of cognitive performance. The dissociation between amyloid and glial/axonal markers likely reflects different stages of cognitive decline, with GFAP and pTau181 indicating clinically relevant downstream neurodegeneration.

By contrast, speech analysis showed limited feasibility, with low uptake, technical challenges, and weak associations with cognitive and biomarker measures^34,35^. Although some verbal fluency metrics aligned with established neuropsychological findings, the multidimensional structure of speech-derived features and limited user acceptance currently restrict their utility for routine community-based detection.

Overall, integrating subjective, cognitive and biological measures provided the clearest differentiation of subgroups. This conceptual approach forms the basis of an adaptive detection framework, where the depth of assessment is tailored to individual profiles. The exploratory clusters generated in the current study offer a preliminary structure for such a model, which will be further refined and validated in future work. Textbox 1 (Theoretical Adaptive Community Case-Finding Model) provides an outline of how this framework could be operationalized in practice.

Despite many participants reporting discussions with their GPs, only seven were formally referred to the memory clinic, highlighting persistent challenges in linking community-based detection to clinical follow-up and underscoring the need for stronger integration with primary care.

### Comparison with prior work

The results align with evidence identifying subjective cognitive decline as a risk marker for future impairment and extend prior work by showing that non-medical community settings can feasibly integrate digital and biomarker-based testing in a cohort of 470 older adults^41,42^. The strongest subgroup differentiation was driven by working memory and learning—domains known to be among the most sensitive early markers in traditional neuropsychological batteries^43,44^. In line with findings that combine brief informant questionnaires (e.g., AD8) with word-list learning enhances predictive value, our data suggest that digital working memory tasks may serve a similar function^36,45^. Because no clinical gold standard was available, these findings should be interpreted conceptually rather than diagnostically.

Biomarker patterns are consistent with evidence indicating that multimarker combinations involving pTau181, GFAP and NfL provide stronger discrimination than single markers^22^. By contrast, plasma Aβ measures remain operationally fragile outside controlled laboratory settings, showing high pre-analytical sensitivity to temperature, time, and handling^46^. We therefore consider p-tau181 and GFAP as the most promising candidates for further evaluation within community-oriented multimodal detection frameworks, although their utility for guiding clinical decision-making requires prospective validation^47,48^.

### Strengths

The study has several important strengths. Its cluster-randomized design across 16 senior centers minimized contamination and ensured pragmatic implementation. The innovative integration of multimodal assessments combining questionnaire data, digital cognition, biomarkers and speech reflect a unique contribution to the field. External validity was enhanced by recruiting participants through senior centers in close collaboration with Johanniter-Unfall-Hilfe e.V., a major non-profit provider of health and elder care services. Finally, engagement was high, with strong participation rates and consistently positive feedback from participants.

### Limitations

Despite these strengths, several limitations must be considered. The sample was demographically skewed, with overrepresentation of women and individuals with tertiary education, which limits the study’s generalizability to more diverse populations. The study was conducted exclusively in a single metropolitan area (Munich), which restricts its external validity further. The lack of a gold standard for outcomes restricts the external validation of the detection measures. Additionally, the limited participation of general practitioners constrained our ability to evaluate how community-based detection could be linked to clinical assessment and follow-up. This suggests that the engagement of GPs could pose a structural barrier to the implementation of community assessments within real-world care pathways. It also highlights the need for studies that explicitly examine how community assessments can be integrated with primary care. Furthermore, the biomarker and speech analyses were exploratory in nature and should be interpreted with caution. Finally, given that senior centers could differ systematically in terms of participant engagement and characteristics, potential cluster-level effects may have influenced the outcomes.

### Implications

Overall, this study demonstrates that innovative, community-based detection strategies are both feasible and well accepted, and that digital and blood-based biomarkers can, in principle, be integrated into everyday care environments outside specialized memory clinics. However, the findings also reveal a critical gap between detection and clinical follow-up: while participation was high, referral to general practitioners remained limited, and few participants underwent formal diagnostic evaluation. Their true preventive potential will depend on system-level integration—particularly reimbursement mechanisms, GP engagement, and clear referral pathways that translate early detection into personalized and sustained care. The value of community-based detection ultimately depends not only on identifying individuals at increased risk but also on improving patient-relevant outcomes, including timely diagnostic clarification, access to counselling and preventive interventions, maintenance of independence, and quality of life.

### Future directions

Future research should validate these findings in more diverse and rural populations and evaluate the added value of informant reports, which show closer alignment with objective impairment (1) and may reduce false positives^17,39^. It remains unclear why GPs perform limited dementia-specific diagnostic work despite guideline familiarity (45, 46), with possible explanations including time constraints, reimbursement barriers, diagnostic uncertainty and concern about early labeling^49–51^. Community detection alone is insufficient without coordinated downstream diagnostic action. Co-managed models linking community providers and GPs may bridge this gap.

Longitudinal validation of multimodal clusters and cost-effectiveness analyses will be essential for establishing practical value^12^. Future adaptations should prioritize simplicity, feedback integration and referral mechanisms that align with primary care workflows. Ultimately, early detection must translate into personally meaningful outcomes for older adults and their families^52^. This includes supporting informed decision-making, promoting access to timely diagnostic evaluation and care, and enabling individuals to engage in preventive actions while autonomy and daily functioning can still be preserved. Community-based detection can serve as a first step in this process, but only if it is embedded within coordinated pathways that ensure continuity from screening to meaningful clinical follow-up.

## Supplementary Information

Supplementary Information.

## Data Availability

The datasets generated and analysed during the current study are not publicly available due to privacy and ethical restrictions but are available from the corresponding author (CK) on reasonable request.

## Abbreviations

AD: Alzheimer’s disease
ANOVA: Analysis of Variance
Aβ: Amyloid-beta
Aβ1-40: Amyloid-beta 1–40
Aβ1-42: Amyloid-beta 1–42
Aβ1-42/1–40: Amyloid-beta 42/40 ratio
BBBM: Blood biomarkers
CI: Confidence interval
EFA: Exploratory factor analysis
FDR: False discovery rate
GFAP: Glial fibrillary acidic protein
GP: General practitioner
IDN RT: Identification reaction time
KMO: Kaiser–Meyer–Olkin statistic
LearnWM: Learning and working memory composite (One card learning + One back)
MCI: Mild cognitive impairment
MMSE: Mini-mental state examination
MoCA: Montreal cognitive assessment
NfL: Neurofilament light chain
ONB: One back
OCL: One card learning
PC: Principal component
PCA: Principal component analysis
pTau181: Phosphorylated tau at threonine 181
PsyAtt: Psychomotor speed and attention composite (Detection + Identification)
RT: Reaction time
SCD-Q: Subjective cognitive decline questionnaire
SB-C: Speech biomarker for cognition
SD: Standard deviation
SEM: Standard error of the mean
