# Supplementary material for: Adaptive pathways for multimodal community-based detection of cognitive impairment: the CogScreen I study
Source: Sci Rep. 2026 Jul 23;16:23072. doi: 10.1038/s41598-026-61868-x (PMC13392269; doi:10.1038/s41598-026-61868-x)
Supplement: Supplementary file 1 — Supplementary Information. [file 41598_2026_61868_MOESM1_ESM.docx]

# **Adaptive Pathways for Multimodal Community-Based Detection of Cognitive Impairment: The CogScreen I Study**

**This Supplement contains:**

**Table S1**: Overview of Variables, Types, and Non-Missing Counts

**Table S2:** SCD-Q Item Descriptive Statistics (Mean % endorsement, SD)

**Figure S1**: Scree plot of factor analysis for self-report questionnaire

**Table S3:** Descriptive statistics for digital cognitive tests

**Figure S2:** Scree plot of factor analysis for digital cognitive tests

**Figure S3:** Scree plot of exploratory factor analysis (EFA) of blood-based biomarkers (BBBMs).

**Table S4:** Descriptive statistics for Speech-derived variables

**Figure S4:** Scree plot of factor analysis for speech analysis

**Table S5:** Correlation of automated speech analysis with SCD-Q total score, BBBM and digital cognitive tests

**Table S1**: Overview of Variables, Types, and Non-Missing Counts

| Variable | Function | Type | N |
| --- | --- | --- | --- |
| ID | Participant identifier | Categorical (string) | 473 |
| Age | Participant age in years at assessment | Continuous | 470 |
| Sex | Self-reported biological sex | Categorical | 471 |
| Education | Total years of formal education | Continuous | 470 |
| Digital cognitive test (Cognigram) | | | |
| OneBack Accuracy, RawAccuracy, LearnWMStdScr | Working memory performance | Continuous | 385 |
| OneCardLearning Accuracy, RawAccuracy, LearnWMStdScr | Learning and memory | Continuous | 385 |
| Detection Accuracy, RawAccuracy, LearnWMStdScr | Basic attention / detection accuracy | Continuous | 362 |
| Identification Accuracy, RawAccuracy | Visual identification accuracy | Continuous | 384 |
| Detection PsyAttStdScr, RawRT, SessionDuration | Detection speed | Continuous | 385 |
| Identification PsyAttStdScr, RawRT | Identification speed | Continuous | 367 |
| OneBack PsyAttStdScr, OneCardLearning PsyAttStdScr | Psychomotor attention | Continuous | 291 |
| Self-Report Questionnaire (SCD-Q) | | | |
| scdq_a Do you perceive memory or cognitive difficulties? | Introductory metacognition items | Dichotomous | 469 |
| scdq_b Would you ask a doctor about these difficulties? | Introductory metacognition items | Dichotomous | 466 |
| scdq_c Has your memory or cognition declined in the last two years? | Introductory metacognition items | Dichotomous | 469 |
| scdq_1 I find it more difficult to learn new phone numbers. | Memory domain | Dichotomous | 468 |
| scdq_2 It is harder for me to find personal belongings. | Other | Dichotomous | 468 |
| scdq_3 It is harder for me to describe the plot of movies. | Language domain | Dichotomous | 469 |
| scdq_4 It is harder for me to remember doctor's appointments. | Memory domain | Dichotomous | 470 |
| scdq_5 I find it more difficult to follow the plot of a book. | Language domain | Dichotomous | 467 |
| scdq_6 I have more difficulty remembering details of a recent family event. | Memory domain | Dichotomous | 470 |
| scdq_7 It is harder for me to remember the outcome of a recent sports event. | Memory domain | Dichotomous | 470 |
| scdq_8 It is harder for me to remember amounts of money. | Memory domain | Dichotomous | 470 |
| scdq_9 It is harder for me to remember details of a conversation. | Memory domain | Dichotomous | 469 |
| scdq_10 It is harder for me to remember things without using strategies. | Memory domain | Dichotomous | 469 |
| scdq_11 It is harder for me to remember the details of recent news. | Memory domain | Dichotomous | 466 |
| scdq_12 It is harder for me to remember the names of famous people. | Memory domain | Dichotomous | 466 |
| scdq_13 It is harder for me to remember the names of people I recently met. | Memory domain | Dichotomous | 468 |
| scdq_14 It is harder for me to remember street and place names. | Memory domain | Dichotomous | 467 |
| scdq_15 It is harder for me to find the word I want to use. | Language domain | Dichotomous | 468 |
| scdq_16 I find it more difficult to understand things when first said. | Language domain | Dichotomous | 466 |
| scdq_17 It is harder for me to remember the names of places I recently visited. | Memory domain | Dichotomous | 467 |
| scdq_18 It is harder for me to concentrate on what I am doing. | Executive / attention domain | Dichotomous | 464 |
| scdq_19 I am worse at planning things that are not routine. | Executive / attention domain | Dichotomous | 467 |
| scdq_20 It is harder for me to use electronic devices. | Executive / attention domain | Dichotomous | 466 |
| scdq_21 I find it more difficult to start new or unfamiliar things. | Executive / attention domain | Dichotomous | 467 |
| scdq_22 I find it more difficult to start new or unfamiliar things. | Executive / attention domain | Dichotomous | 467 |
| scdq_23 I find it more difficult to do mental arithmetic. | Executive / attention domain | Dichotomous | 467 |
| scdq_24 I find it more difficult to do more than one thing at a time. | Executive / attention domain | Discrete (0 – 24) | 461 |
| SCDQ Total Score | Global SCD-Q summary score | float64 | 436 |
| Speech analysis | | | |
| Semantic fluency: total correct | Semantic fluency output | Continuous | 473 |
| Semantic fluency: mean word frequency | Lexical properties | Continuous | 473 |
| Semantic fluency: mean semantic distance | Semantic similarity | Continuous | 66 |
| Temporal clustering: mean cluster size | Temporal dynamics | Continuous | 66 |
| Temporal clustering: number of switches | Temporal dynamics | Continuous | 66 |
| Temporal clustering: mean intra-cluster transition time | Temporal dynamics | Continuous | 66 |
| Temporal clustering: mean inter-cluster transition time | Temporal dynamics | Continuous | 66 |
| Semantic clustering: mean cluster size | Semantic structure | Continuous | 66 |
| Semantic clustering: number of switches | Semantic structure | Continuous | 66 |
| Semantic clustering: mean inter-cluster similarity | Semantic structure | Continuous | 66 |
| Semantic clustering: mean intra-cluster similarity | Semantic structure | Continuous | 66 |
| Semantic clustering: mean consecutive similarity | Semantic structure | Continuous | 66 |
| Semantic clustering: consecutive similarity (bin 1) | Semantic structure | Continuous | 66 |
| Semantic clustering: consecutive similarity (bin 2) | Semantic structure | Continuous | 66 |
| Semantic clustering: consecutive similarity (bin 3) | Semantic structure | Continuous | 66 |
| Semantic clustering: consecutive similarity (bin 4) | Semantic structure | Continuous | 66 |
| Semantic clustering: consecutive similarity (bin 5) | Semantic structure | Continuous | 66 |
| Semantic clustering: consecutive similarity (bin 6) | Semantic structure | Continuous | 66 |
| Composite Semantic Distance Feature: mean across bins | Semantic similarity | Continuous | 66 |
| Blood-based Biomarkers | | | |
| Amyloid-β 1–42 (pg/ml) | Amyloid pathology | continuous | 220 |
| Amyloid-β 1–40 (ng/ml) | Amyloid pathology | continuous | 220 |
| Aβ 1–42/1–40 ratio (dimensionless) | Amyloid pathology | continuous | 220 |
| Phospho-Tau 181 (pTau181) (pg/ml) | Tau pathology | continuous | 220 |
| Glial Fibrillary Acidic Protein (GFAP) (ng/ml) | Astroglial activation | continuous | 220 |
| Neurofilament Light Chain (NfL) (pg/ml) | Neurodegeneration / axonal injury | continuous | 220 |
| Apolipoprotein E4 (ApoE4) (mg/ml) | Lipid and amyloid metabolism | continuous | 220 |

**Table S2**: SCD-Q Item Descriptive Statistics (Mean % endorsement, SD)

| Item | Mean | SD | Item | Mean | SD |
| --- | --- | --- | --- | --- | --- |
| scdq_1 | 47.11 | 0.500 | scdq_13 | 51.93 | 0.500 |
| scdq_2 | 30.04 | 0.459 | scdq_14 | 42.06 | 0.494 |
| scdq_3 | 21.32 | 0.410 | scdq_15 | 54.72 | 0.498 |
| scdq_4 | 21.11 | 0.409 | scdq_16 | 22.37 | 0.417 |
| scdq_5 | 19.06 | 0.393 | scdq_17 | 32.76 | 0.470 |
| scdq_6 | 20.68 | 0.405 | scdq_18 | 20.13 | 0.401 |
| scdq_7 | 30.28 | 0.460 | scdq_19 | 23.87 | 0.427 |
| scdq_8 | 16.20 | 0.369 | scdq_20 | 29.89 | 0.458 |
| scdq_9 | 39.23 | 0.489 | scdq_21 | 38.41 | 0.487 |
| scdq_10 | 55.65 | 0.497 | scdq_22 | 15.12 | 0.359 |
| scdq_11 | 33.76 | 0.473 | scdq_23 | 31.83 | 0.466 |
| scdq_12 | 53.55 | 0.499 | scdq_24 | 35.65 | 0.479 |

**
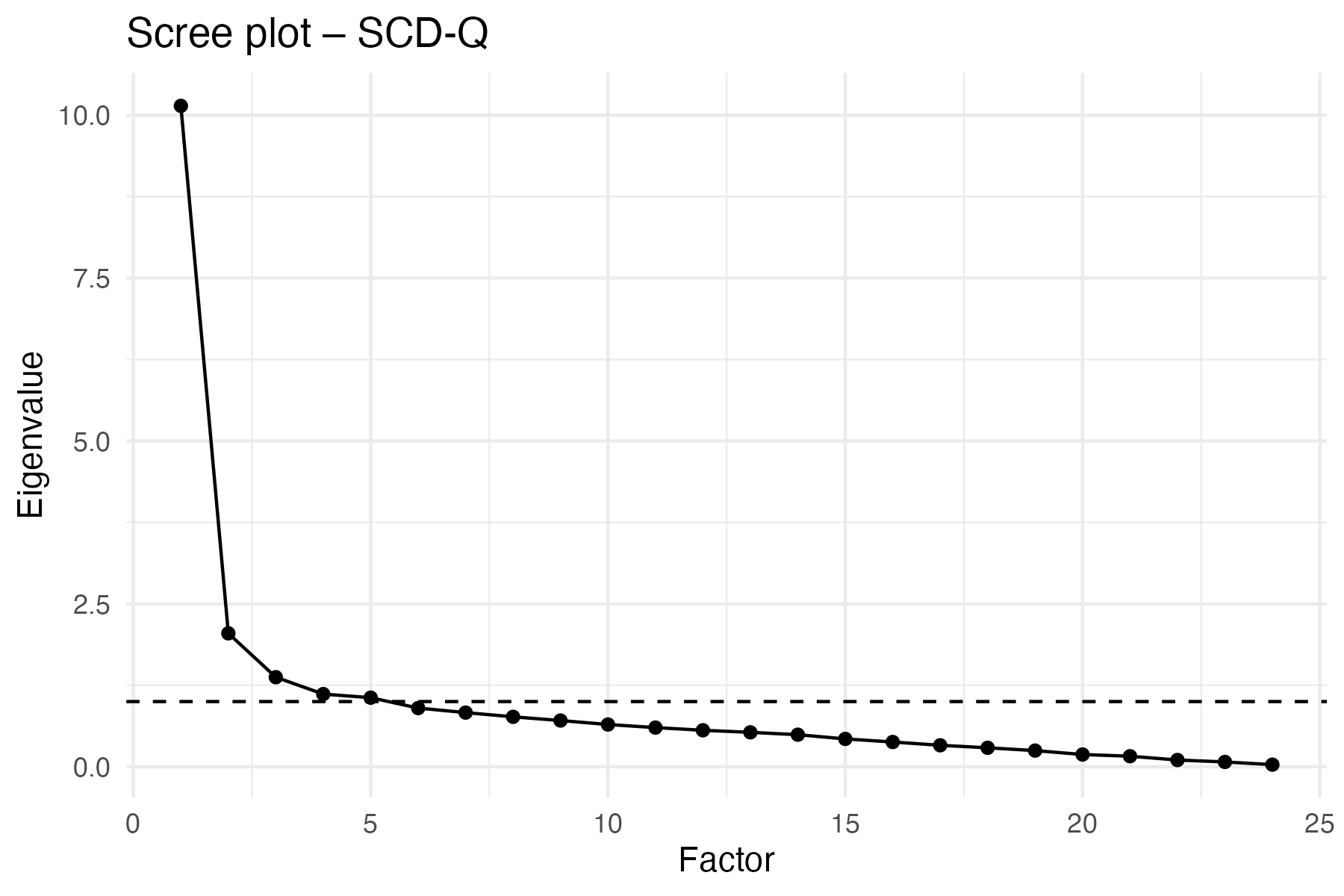
Figure S1:** Scree plot of factor analysis of self-report questionnaire

**Legend Figure S1:** The Kaiser–Meyer–Olkin (KMO) measure of sampling adequacy was .70, indicating acceptable suitability for factor analysis. Bartlett’s test of sphericity was highly significant, χ²(276) = 7,529.41, p < 2×10⁻¹⁶, confirming that the correlation matrix was factorable. Exploratory factor analysis (maximum-likelihood, oblimin rotation) suggested a multifactorial structure, with five factors retained based on the Kaiser criterion. Several SCD-Q items showed salient loadings (≥ .40) across different factors, most prominently items related to memory (e.g., scdq_9, scdq_10, scdq_11), language (e.g., scdq_12–14, scdq_17), and executive functioning (e.g., scdq_16, scdq_20–22). This pattern indicates that subjective cognitive decline as measured by the SCD-Q reflects a heterogeneous set of perceived difficulties rather than a single underlying dimension.

**Table S3:** Descriptive statistics for Digital cognitive tests (n, Mean, SD, Min, Max)

| Variable | Unit | N | Mean | SD | Min | Max |
| --- | --- | --- | --- | --- | --- | --- |
| Detection accuracy | Arcsine-sqrt proportion | 291 | 1.475 | 0.132 | 0.961 | 1.571 |
| Detection accuracy | % correct | 291 | 0.975 | 0.048 | 0.672 | 1.000 |
| Detection reaction time | ms | 291 | 605.520 | 287.860 | 261.314 | 2,101.457 |
| Detection session duration | ms | 291 | 967,671.251 | 246,587.944 | 422,943.000 | 2,369,095.000 |
| Identification accuracy | Arcsine-sqrt proportion | 290 | 1.466 | 0.143 | 1.029 | 1.571 |
| Identification accuracy | % correct | 290 | 0.970 | 0.052 | 0.734 | 1.000 |
| Identification reaction time | ms | 290 | 766.776 | 178.308 | 456.367 | 1,571.600 |
| One-back accuracy | % correct | 283 | 0.908 | 0.109 | 0.261 | 1.000 |
| One-back reaction time | ms | 283 | 1,218.468 | 328.826 | 675.129 | 2,289.742 |
| One-card learning accuracy | % correct | 286 | 0.632 | 0.099 | 0.294 | 0.880 |
| One-card learning reaction time | ms | 286 | 1,808.409 | 689.600 | 826.367 | 8,165.536 |
| Detection psychomotor/attention | T-score | 291 | 83.280 | 12.942 | 37.331 | 111.064 |
| Identification psychomotor/attention | T-score | 290 | 83.439 | 12.679 | 39.366 | 111.064 |
| One-back psychomotor/attention | T-score | 283 | 83.760 | 12.719 | 39.366 | 121.344 |
| One-card learning psychomotor/attention | T-score | 286 | 83.621 | 12.604 | 39.366 | 111.064 |

**Legend Table S4:** Note Values are on original scales. Placeholder -99 treated as NA, Raw accuracy values represent the proportion of correct responses (0–1), reported here as percentages for interpretability. Transformed accuracy values (*_accuracy) are arcsine-square-root transformed proportions, dimensionless, ranging from 0 to 1.571. Reaction times and session durations are given in milliseconds (ms), with session duration additionally reported as minutes. Standardized scores (*_std_scr) are T-scores (M = 100, SD = 15).

**
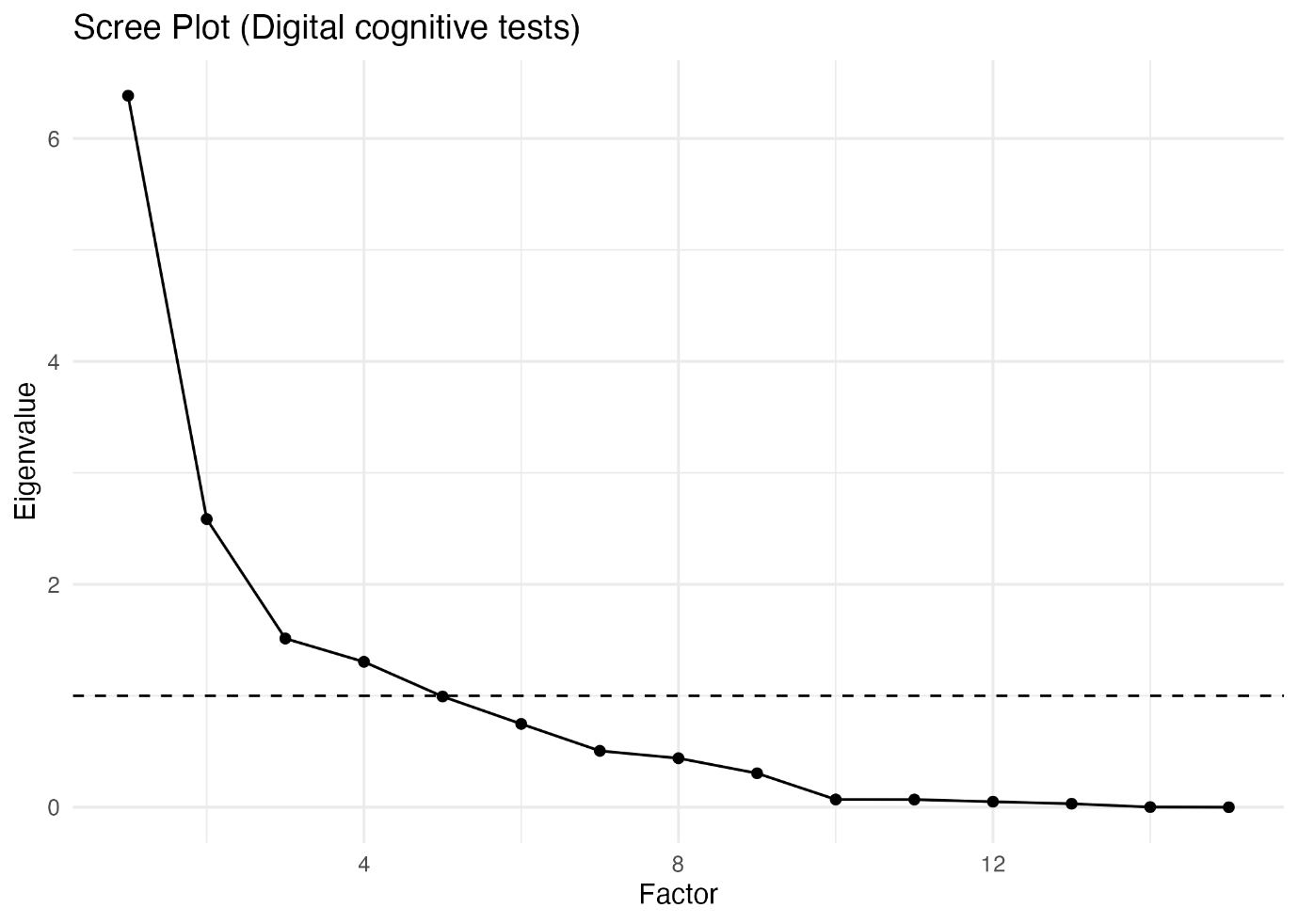
Figure S2:** Scree plot of factor analysis of digital cognitive subtests

**Legend Figure S2:** The Kaiser–Meyer–Olkin (KMO) measure of sampling adequacy was .50, indicating only marginal suitability for factor analysis. Bartlett’s test of sphericity, however, was highly significant, χ²(105) = 14,699.82, p < 2 × 10⁻¹⁶, supporting the factorability of the correlation matrix. Exploratory factor analysis (maximum-likelihood, oblimin rotation) suggested a multifactorial solution, with four factors retained under the Kaiser criterion. Salient loadings (≥ .40) emerged for multiple variables: psychometric T-scores (e.g., detection and identification composite scores) and reaction times (e.g., detection, identification, one-back, and one-card learning) showed distinct clustering, while accuracy indices (raw and transformed) loaded on separate factors. This pattern indicates that the digital cognitive battery reflects heterogeneous but partly correlated domains, including processing speed, accuracy, and standardized attention/memory indices. Overall, the relatively low KMO value suggests weak shared variance, and results should therefore be interpreted with caution.

**
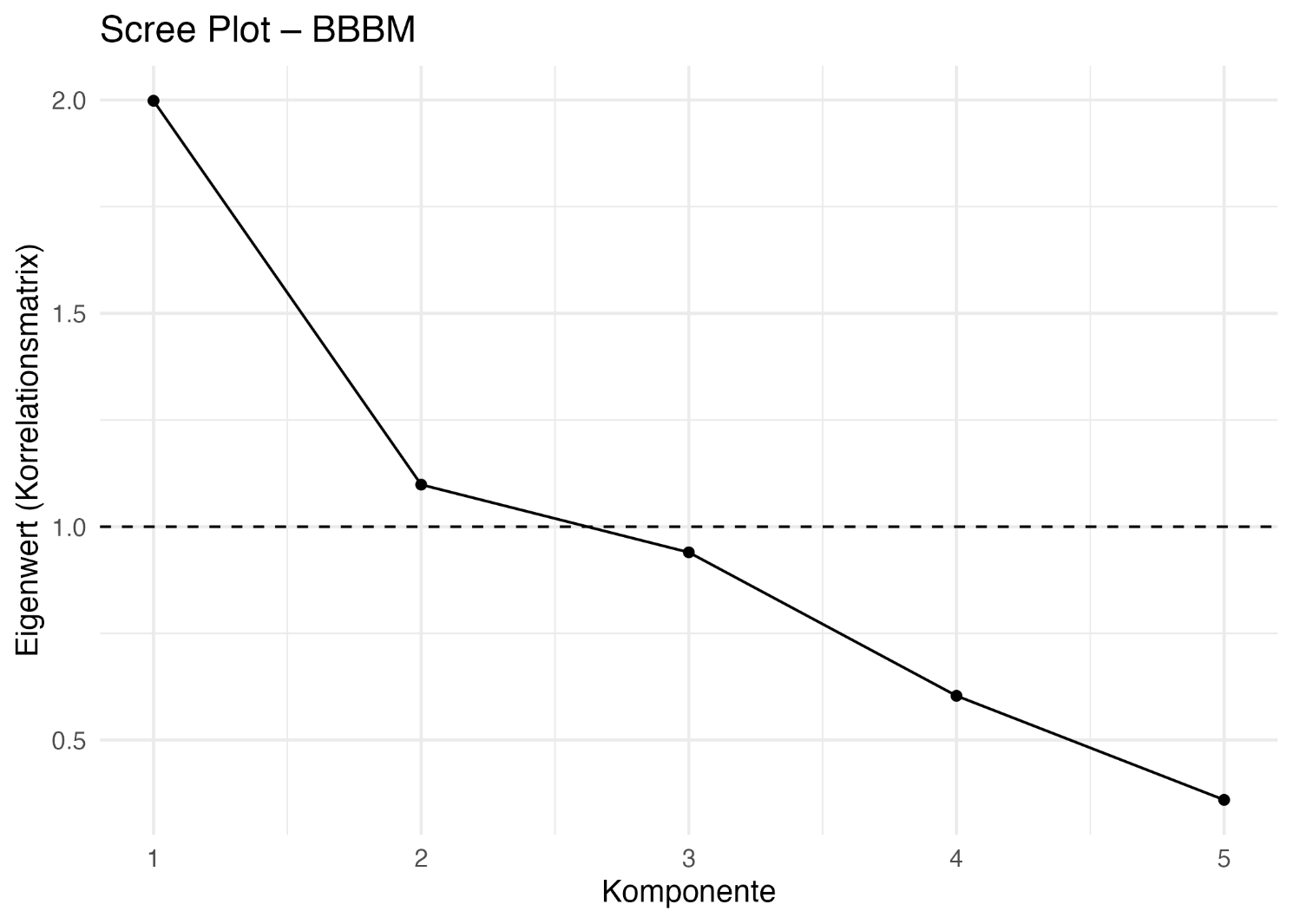
Figure S3:** Scree plot of exploratory factor analysis (EFA) of blood-based biomarkers (BBBMs).

**Legend Figure S4:**   Exploratory factor analysis (maximum likelihood, oblimin rotation) of plasma biomarkers. Aβ42/Aβ40 ratio was used as provided (dimensionless). The Kaiser–Meyer–Olkin (KMO) (eigenvalue > 1) measure of sampling adequacy was 0.63, indicating moderate suitability for factor analysis. Bartlett’s test of sphericity was highly significant, χ²(10) = 376.86, p < 7.9×10⁻⁷⁵, confirming factorability of the correlation matrix. Three factors were retained based on the Kaiser criterion (eigenvalues > 1). Factor 1 was defined by GFAP (loading = .88), NFL (.69), and pTau181 (.56), reflecting markers of neurodegeneration and glial pathology. Factor 2 was dominated by Aβ40 (.81) and negatively by Aβ42/Aβ40 ratio (–.63), representing amyloid-related variance. Factor 3 was strongly associated with Aβ42 (.95) and Aβ40 (.35), capturing additional amyloid variance. Communalities (h²) indicated that most markers shared a substantial proportion of variance with the latent factors (e.g., Aβ42 h² = .995; GFAP h² = .769). In contrast, APOE4 status showed almost no shared variance (h² = .011), reflecting its categorical nature and weak factor representation.

**Table S4:** Descriptive statistics for Speech-derived variables (n, Mean, SD, Min, Max)

| **Section** | **Variable** | **Unit** | **N** | **Mean** | **SD** | **Min** | **Max** |
| --- | --- | --- | --- | --- | --- | --- | --- |
| Semantic Clustering | meanclustersize | n | 59 | 3.43 | 1.265 | 1.5 | 8.5 |
|  | mean consecutive similarity | 0–1 | 59 | 0.47 | 0.033 | 0.39 | 0.55 |
|  | inter cluster similarity | 0–1 | 59 | 0.487 | 0.087 | 0.3 | 0.74 |
|  | intra cluster similarity | 0–1 | 58 | 0.493 | 0.042 | 0.42 | 0.6 |
|  | n switches | n | 59 | 5.712 | 1.992 | 1.0 | 11.0 |
| Semantic – Time Bins | consec sim bin1 | 0–1 | 59 | 0.483 | 0.059 | 0.34 | 0.63 |
|  | consec sim bin2 | 0–1 | 58 | 0.452 | 0.17 | 0.0 | 1.0 |
|  | consec sim bin3 | 0–1 | 56 | 0.405 | 0.206 | 0.0 | 0.74 |
|  | consec sim bin4 | 0–1 | 55 | 0.432 | 0.207 | 0.0 | 0.77 |
|  | consec sim bin5 | 0–1 | 54 | 0.453 | 0.215 | 0.0 | 1.0 |
|  | consec sim bin6 | 0–1 | 50 | 0.408 | 0.27 | 0.0 | 1.0 |
| Temporal Measures | mean utterance distance | ms | 66 | 930.629 | 931.04 | 0.0 | 3954.55 |
|  | temporal cluster size | ms | 66 | 1644.022 | 2181.139 | 10.0 | 8333.33 |
|  | inter cluster TT | ms | 66 | 1416.177 | 2413.264 | 0.0 | 10366.67 |
|  | intra cluster TT | ms | 66 | 261.832 | 486.656 | 0.0 | 2086.11 |
|  | temporal n switches | n | 66 | 4.545 | 2.069 | 0.0 | 9.0 |
| Output / Lexical | correct count animals | n | 66 | 20.409 | 5.935 | 1.0 | 34.0 |
|  | word frequency mean | dimensionless | 66 | 2549.057 | 1640.166 | 293.0 | 3943.08 |

**Legend Table S4:** Speech-derived variables were obtained from the Semantic Verbal Fluency task (category “animals”) following the ki:e SB-C protocol. Measures include overall output (number of correct responses, mean word frequency), semantic and temporal clustering metrics, and similarity indices. Bin variables (semantic clustering mean consecutive similarity bin1–6) capture mean semantic similarity of consecutive responses within successive 10-second intervals of the 1-minute task, providing a temporal profile of clustering behavior. Unless otherwise noted, values are dimensionless similarity scores (0–1); temporal measures are in milliseconds (ms), and switch variables are simple counts.

**Figure S4:** Scree plot of principal component analysis (PCA) for automated speech analysis

**Legend Figure S3:** Scree plot of principal component analysis (PCA) of speech-derived variables.

The Kaiser–Meyer–Olkin (KMO) measure of sampling adequacy was **0.56**, indicating mediocre adequacy. Bartlett’s test of sphericity was significant, **χ²(153) = 335.8, p < .001**, confirming factorability of the correlation matrix. According to the scree plot and the Kaiser criterion, **seven factors** were extracted.

- **Factor 1**: strong loadings on *semantic consecutive similarity* (overall and bin4) and *semantic intra cluster similarity* → captures **semantic similarity within clusters**.
- **Factor 2**: high loading of *correct count animals* → reflects **overall task performance / correct responses**.
- **Factor 3**: Semantic clustering (↑) vs. switching (↓) dimension
- **Factor 4**: *Utterance distance and temporal transition dynamics*
- **Factor 5**: *semantic consecutive similarity bin2* and *bin5* → a **bin-specific semantic similarity** factor.
- **Factor 6**: *temporal cluster size* → reflects **temporal cluster size**.
- **Factor 7**: *semantic consecutive similarity bin1* (negative) → a **bin-specific effect**.

Together, these seven factors explained the majority of variance across the **18 included speech-derived measures**, suggesting a multifaceted latent structure underlying **correct responses, semantic/temporal clustering, similarity measures, and transition times** in verbal fluency.

**Table S5: Partial correlations: Speech outcomes vs Predictors (adjusted for age, sex)**

| Predictor (X) | Speech (Y) | Partial r | p (uncorr.) | p (FDR) | n |
| --- | --- | --- | --- | --- | --- |
| pTau181 | Fluency correct | 0.396323489 | 0.06117742 | 0.9185022 | 23 |
|  | Intra-cluster TT | -0.290011011 | 0.17948478 | 0.9185022 | 23 |
|  | Semantic consec sim | -0.058441975 | 0.7911066 | 0.9185022 | 23 |
|  | Semantic cluster size | -0.054881053 | 0.80358539 | 0.9185022 | 23 |
|  | Semantic switches | -0.049600644 | 0.82217477 | 0.9185022 | 23 |
|  | Utterance distance | -0.266317625 | 0.21932625 | 0.9185022 | 23 |
| Abeta1-42/1-40 ratio | Fluency correct | 0.161667499 | 0.46114562 | 0.9185022 | 23 |
|  | Intra-cluster TT | 0.043488401 | 0.84380802 | 0.9185022 | 23 |
|  | Semantic cluster size | 0.077784904 | 0.724257 | 0.9185022 | 23 |
|  | Semantic consec sim | 0.080924263 | 0.71357491 | 0.9185022 | 23 |
|  | Semantic switches | 0.091726604 | 0.67722501 | 0.9185022 | 23 |
|  | Utterance distance | 0.060325992 | 0.78452412 | 0.9185022 | 23 |
| GFAP | Fluency correct | -0.103993035 | 0.63678095 | 0.9185022 | 23 |
|  | Intra-cluster TT | 0.042381359 | 0.84773839 | 0.9185022 | 23 |
|  | Semantic cluster size | -0.034525463 | 0.87572506 | 0.9185022 | 23 |
|  | Utterance distance | 0.086803631 | 0.69371022 | 0.9185022 | 23 |
| NfL | Fluency correct | -0.092123349 | 0.67590255 | 0.9185022 | 23 |
|  | Intra-cluster TT | -0.081870208 | 0.71036627 | 0.9185022 | 23 |
|  | Semantic consec sim | -0.058211224 | 0.79191377 | 0.9185022 | 23 |
|  | Semantic cluster size | -0.06115466 | 0.78163335 | 0.9185022 | 23 |
|  | Semantic switches | -0.057061707 | 0.79593786 | 0.9185022 | 23 |
|  | Utterance distance | -0.018135501 | 0.93454258 | 0.9521755 | 23 |
| SCD-Q total | Intra-cluster TT | -0.140413182 | 0.26844123 | 0.9185022 | 64 |
|  | Fluency correct | -0.135521947 | 0.2856366 | 0.9185022 | 64 |
|  | Semantic consec sim | -0.090902857 | 0.47499785 | 0.9185022 | 64 |
|  | Semantic cluster size | -0.091619889 | 0.4715068 | 0.9185022 | 64 |
|  | Semantic switches | -0.082328453 | 0.51779641 | 0.9185022 | 64 |
|  | Utterance distance | -0.261385579 | 0.0369522 | 0.9185022 | 64 |
| OCL Accuracy | Semantic cluster size | -0.124032392 | 0.36694449 | 0.9185022 | 55 |
|  | Semantic consec sim | -0.12718808 | 0.35478275 | 0.9185022 | 55 |
|  | Semantic switches | -0.129772887 | 0.34500764 | 0.9185022 | 55 |
|  | Utterance distance | 0.213093561 | 0.11827705 | 0.9185022 | 55 |
| ONB Accuracy | Intra-cluster TT | -0.10447325 | 0.44780531 | 0.9185022 | 55 |
|  | Fluency correct | -0.048894548 | 0.72296792 | 0.9185022 | 55 |
|  | Semantic cluster size | 0.050463035 | 0.71445374 | 0.9185022 | 55 |
|  | Semantic consec sim | 0.046542044 | 0.73580037 | 0.9185022 | 55 |
|  | Semantic switches | 0.035941977 | 0.79446688 | 0.9185022 | 55 |
|  | Utterance distance | -0.171976628 | 0.20929853 | 0.9185022 | 55 |
| IDN RT | Fluency correct | -0.116052679 | 0.3943504 | 0.9185022 | 56 |
|  | Semantic cluster size | 0.123725214 | 0.36361964 | 0.9185022 | 56 |
|  | Semantic consec sim | 0.12623574 | 0.35388946 | 0.9185022 | 56 |
|  | Semantic switches | 0.118027796 | 0.38629715 | 0.9185022 | 56 |
|  | Utterance distance | 0.154055161 | 0.256952 | 0.9185022 | 56 |
| DET RT | Fluency correct | 0.212018138 | 0.12018958 | 0.9185022 | 55 |
|  | Semantic cluster size | 0.066215535 | 0.63100271 | 0.9185022 | 55 |
|  | Semantic consec sim | 0.058760208 | 0.67000831 | 0.9185022 | 55 |
|  | Semantic switches | 0.056133647 | 0.68396569 | 0.9185022 | 55 |

**Legend Table S5:** r = partial correlation; p (uncorr.) = uncorrected p-value; p (FDR) = Benjamini–Hochberg adjusted p-value; * < .05, ** < .01, *** < .001. OCL Accuracy = accuracy in the One Card Learning task. ONB Accuracy = accuracy in the One Back task. IDN RT = reaction time in the Identification task. DET RT = reaction time in the Detection task.
